# Comparison of Estimating Equations for the Prediction of Glomerular Filtration Rate in Kidney Donors before and after Kidney Donation

**DOI:** 10.1371/journal.pone.0060720

**Published:** 2013-04-09

**Authors:** Byung Ha Chung, Jee Hyun Yu, Hyuk Jin Cho, Ji-Il Kim, In Sung Moon, Cheol Whee Park, Chul Woo Yang, Yong-Soo Kim, Bum Soon Choi

**Affiliations:** 1 Transplant research center, Seoul St. Mary’s Hospital, College of Medicine, The Catholic University of Korea, Seoul, Korea; 2 Division of Nephrology, Department of Internal Medicine, Seoul St. Mary’s Hospital, College of Medicine, The Catholic University of Korea, Seoul, Korea; 3 Department of Urology, Seoul St. Mary’s Hospital, College of Medicine, The Catholic University of Korea, Seoul, Korea; 4 Department of Surgery, Seoul St. Mary’s Hospital, College of Medicine, The Catholic University of Korea, Seoul, Korea; The University of Manchester, United Kingdom

## Abstract

The aim of this study is to investigate the usefulness of the GFR-estimating equations to predict renal function in kidney donors before and after transplantation. We compared the performance of 24-hour-urine–based creatinine clearance (24 hr urine-CrCl), the Cockcroft-Gault formula (eGFR_CG_), the Modification of Diet in Renal Disease equation (eGFR_MDRD_), and the Chronic Kidney Disease Epidemiology Collaboration equation (eGFR_CKD-EPI_) with technetium-diethylenetriaminepentaacetic acid (^99m^Tc-DTPA) clearance (mGFR) in 207 potential kidney donors and 108 uninephric donors. Before donation, eGFR_CKD-EPI_ showed minimal bias and did not show a significant difference from mGFR (P = 0.65, respectively) while 24 hr urine-CrCl and eGFR_MDRD_ significantly underestimated mGFR (P<0.001 for each). Precision and accuracy was highest in eGFR_CKD-EPI_ and this better performance was more dominant when renal function is higher than 90 mL·min^−1^·1.73 m^−2^. After kidney donation, eGFR_MDRD_ was superior to other equations in precision and accuracy in contrast to before donation. Within individual analysis, eGFR_MDRD_ showed better performance at post-donation compared to pre-donation, but eGFR_CKD-EPI_ and eGFR_CG_ showed inferior performance at post-donation. In conclusion, eGFR_CKD-EPI_ showed better performance compared to other equations before donation. In a uninephric donor, however, eGFR_MDRD_ is more appropriate for the estimation of renal function than eGFR_CKD-EPI_.

## Introduction

Assessment of renal function is a critical component of donor evaluation in kidney transplantation and regular monitoring of it is recommended for the long-term safety of kidney donors. [1] In many centers, measurement of GFR using the ^125^I-iothalamate GFR or technetium-99 m diethylenetriaminepentaacetic acid (^99m^Tc DTPA) clearance is performed before kidney donation. [2], [3] However, those studies are available only in a limited number of institutions. Moreover, they are not feasible for the post-donation monitoring of GFR in everyday clinical practice. Therefore, creatinine-based GFR estimations have been used as alternatives for the estimation of renal function before and after donation.

The 2 equations most commonly used are the Modification of Diet in Renal Disease (MDRD) Study equation and the Cockcroft-Gault (CG) formula. These formulas have some limitations for use in kidney donor workup, because they were developed based on data from patients with reduced renal function. [4], [5], [6] Recently, the Chronic Kidney Disease Epidemiology Collaboration developed a new equation (CKD-EPI). [7] Its aim was to eliminate the weak point of the MDRD formula and the underestimation of GFR; the data set of the CKD-EPI formula included many participants with normal GFR in the development process. During the validation process in several populations, it has shown greater precision and reliability compared with those of the MDRD formula, especially for subjects with GFR of >60 mL·min^−1^·1.73 m^−2^. [7], [8], [9].

The aim of this study was thus to investigate the performance of each GFR-estimating equations in the prediction of renal function in kidney donors. Second, we intended to determine the usefulness of those equations for the post-donation monitoring of renal function in uninephric donors.

## Materials and Methods

### Patients and Methods

A total of 207 healthy Korean adults who underwent the kidney donor workup at our center between March 2009 and September 2011 were included in this study. Laboratory evaluation included blood urea nitrogen, serum creatinine (Scr), and 24 hour urine-based creatinine clearance (24-hour urine CrCl). Scr values were measured in a single laboratory using a “compensated” IDMS-traceable method (Hitachi Modular P-800; Roche Diagnostics, Germany). GFR was measured (mGFR) by ^99m^Tc DTPA clearance with a single injection technique with a 4-point sampling approach at 10, 30, 180, and 240 minutes after injection, according to the method described by Russel et al. [10] After kidney donation, donors visited an outpatient clinic every 3 months, where blood chemistry examination including Scr was performed for 1 year after kidney transplantation (KT). ^99m^Tc DTPA clearance was performed at around 6 months from KT, as part of the routine follow-up process. Out of 207 patient populations, 108 subjects completed studies after kidney donation, and they were included in the post-donation analysis.

Estimated glomerular filtration rate (eGFR) was calculated using the following equations.

- Creatinine clearance (CrCl) based on 24-hour urine chemistry:

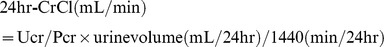

- Cockcroft-Gault method [5]:

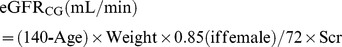

- MDRD formula [11]:

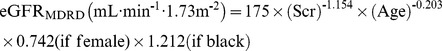

- CKD-EPI equation [7]

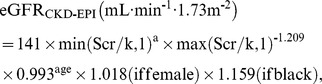
where *k* is 0.7 for women and 0.9 for men, *a* is −0.329 for women and −0.411 for men, *min* indicates the minimum of Scr/kr or 1, and *max* indicates the maximum or Scr/k or 1.

- Body-surface area (BSA) was calculated using the following formulae

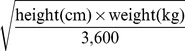



The results of mGFR, 24 hr-CrCl, and eGFR_CG_ were corrected to standard BSA (1.73 m^2^). This study was approved by the Institutional Review Board of Seoul St. Mary’s Hospital (KC12RISI0387).

### Statistical Analysis

Data are presented as mean ± SD or counts and percentages, depending on the data type. For continuous variables, mean values were compared using Oneway ANOVA and Dunnett’s test. The mean difference between equation-based GFR and the measured GFR was used to determine the bias. Pair-wise comparison of the mean difference was performed using the paired *t*-test. The precision of the estimates was determined as SD of the mean difference between mGFR and eGFR. [12] Accuracy-integrating precision and bias was calculated as the percentage of GFR estimates within 10% and 30% of the measured GFR as suggested. [13] McNemar’s test was used to evaluate the degree of accuracy. [14] Comparison of the correlation coefficients was performed using Z-statistics. Moreover, a graphical approach to assess accuracy was carried out according to the Bland-Altman method. [15] Statistical analyses were performed using SPSS software (version 15.0; SPSS Inc., Chicago, IL, USA) and MedCalc 11.2.1.0 (Medcalc, Mariakerke, Belgium). All tests were 2-tailed, and the results were considered significant when the P value was below 0.05.

## Results

### Baseline Characteristics of Patient Population

In the pre-donation analysis, the mean age of donors was 40.4±11.3 years; 87 were male (42.0%). The mean height and weight were 164.0±8.9 cm and 64.0±11.9 kg, respectively. The mean body surface area (BSA) was 1.67±0.17 m^2^; the mean body mass index (BMI) was 23.1±4.4 kg/m^2^. The mean Scr was 0.78±0.16 mg/dL. In the post-donation analysis in 108 uni-nephric donors, the interval from KT to the measurement of GFR and Scr was 7.3±3.9 months. Mean patient age was 39.0±11.5 years; 51 patients were male (47.2%). Scr at the measurement of mGFR was 1.07±0.25 mg/dL.

### Comparison of Each Equation’s Performance to Predict mGFR before Kidney Donation

Table 1 provides overall results for the bias, precision, and accuracy of all equations for the estimation of mGFR in this pre-donation cohort. eGFR_CG_ and eGFR_CKD-EPI_ showed minimal bias (P = 0.99 and P = 0.92 vs. mGFR, respectively), while 24 hr urine-CrCl and eGFR_MDRD_ significantly underestimated mGFR (P<0.001 vs. mGFR in each case). eGFR_CKD-EPI_ showed highest precision (lowest SD of mean bias) among three equations. In addition, the accuracy of eGFR_CKD-EPI_ within 30% of mGFR was 91.8%, which is significantly higher than that of 24 hr urine-CrCl (71.5%) and eGFR_MDRD_ (84.1%) (P<0.001 in each case) and it showed a higher tendency compared with eGFR_CG_ (86.0%) (P = 0.06).

**Table 1:** Comparison of the bias, precision and accuracy in the estimation of mGFR among each equation before kidney donation according to the mGFR level.

mGFR Group(mL/min/1.73 m2)	eGFR	Value(mL/min/1.73 m^2^)Mean±SD	Meandifference tomGFR	Median differenceto mGFR	SD ofmean bias	Accuracy within	
						10% (%)	30% (%)
	^99m^Tc DTPA	110.3±20.7	–	–	–	–	–
	24 hr urine-CrCl	97.4±31.5*	−12.5#,¶,	−13.2(−89.3–119.4)	29.4¶,$	25.1¶	71.5¶
All (n = 207)	eGFR_CG_	109.6±27.9	−0.73** ^,^ $	−2.9 (−52.0−76.7)	22.9	33.3	86.0
	eGFR_MDRD_	100.7±20.4*	−9.6#,¶	−9.0 (−69.1−50.8)	20.8	35.3	84.1¶
	eGFR_CKD-EPI_	108.7±18.0	−1.6** ^,^ $	0.4 (−55.5−45.1)	19.1	40.6	91.8** ^,^ #,$
	^99m^Tc DTPA	116.3±17.7	–	–	–	–	–
	24 hr urine-CrCl	102.0±32.1*	−14.3#,¶	−16.6 (−89.3–119.4)	31.4¶,$	24.9¶	68.0¶
≥90 (n = 170)	eGFR_CG_	114.7±27.5	−1.5** ^,^ $	−5.6 (−52.0–76.7)	24.4¶	30.8¶	84.6¶
	eGFR_MDRD_	103.7±20.1*	−12.5#,¶	−13.0 (−69.1–50.8)	21.0**	33.7	82.2¶
	eGFR_CKD-EPI_	111.8±17.3	−4.5** ^,^ $	−3.7 (−55.5–45.1)	19.1**	42.0	94.7** ^,^ #,$
	^99m^Tc DTPA	83.1±6.4	–	–	–	–	–
	24 hr urine-CrCl	78.7±16.5	−4.5	−7.3 (−38.0–25.6)	15.7	27.0	89.2
<90 (n = 37)	eGFR_CG_	86.0±15.1	2.9¶	4.3 (−24.5–36.5)	13.9	45.9	81.1
	eGFR_MDRD_	86.8±15.1	3.6¶	2.0 (−24.0–43.3)	13.9	43.2	94.6
	eGFR_CKD-EPI_	94.7±14.4*	11.6** ^,^ #	11.2 (−13.3–40.4)	12.6	35.1	81.1

mGFR, measured glomerular filtration rate,^ 99m^Tc DTPA, technetium-diethylenetriamine pentaacetic acid, 24 hr urine-CrCl, creatinine clearance; eGFR_CG_, Cock-Croft Gault; eGFR_MDRD_, Modification of Diet in Renal Disease; eGFR_CKD-EPI_, chronic kidney disease-Epidemiology collaboration.

* P<0.05, vs. mGFR,

** P<0.05 vs. 24 hr urine-CrCl,

# P<0.05 vs. eGFR_CG_,

$ P<0.05, vs. eGFR_MDRD_,

¶ P<0.05 vs eGFR_CKD-EPI._

### Comparison of Each Equation’s Performance According to mGFR Level before Kidney Donation

We analyzed the performance of equations according to renal function (Table 1). In 170 subjects with normal renal function (mGFR ≥90 mL·min^−1^·1.73 m^−2^), both 24 hr urine-CrCl and eGFR_MDRD_ significantly underestimated mGFR (P<0.001 vs. mGFR in each case), but eGFR_CKD-EPI_ showed little bias (P = 0.92). eGFR_CKD-EPI_ showed higher precision and accuracy within 30% of mGFR (P<0.05 in each case) than the other 3 equations as well. In 37 subjects with decreased renal function (mGFR ≤90 mL·min^−1^·1.73 m^−2^), eGFR_CKD-EPI_ significantly overestimated mGFR (P<0.001 vs. mGFR), and 24 hr-urine CrCl, eGFR_CG_ and eGFR_MDRD_ did not show significant bias to mGFR (P = 0.094, P = 0.211 and P = 0.123 vs. mGFR, respectively). In precision and accuracy, no significant differences were detected in any comparisons between equations.

### Comparison of Each Equation’s Performance to Predict mGFR after Kidney Donation

Table 2 provides overall results for the bias, precision, and accuracy of all three equations for the estimation of mGFR in this post-donation cohort. eGFR_CKD-EPI_ showed the least bias as compared with mGFR (P = 1.0 vs. mGFR) as like in pre-donation analysis. In contrast, eGFR_MDRD_ (SD of mean bias: 17.2) showed significantly higher precision as compared to eGFR_CG_ (20.8) and eGFR_CKD-EPI_ (22.9) (P<0.001 in each case). The accuracy within 10% and 30% of mGFR was significantly higher for eGFR_MDRD_ as compared with eGFR_CG_ and eGFR_CKD-EPI_ as well (P<0.05 in each case) (Table 2).

**Table 2:** Comparison of the bias, precision and accuracy in the estimation of mGFR among each equation after kidney donation according to the mGFR level.

mGFR group(mL/min/1.73 m^2^)	eGFR	Value(mL/min/1.73m^2^)Mean±SD	Mean differenceto mGFR	Mediandifference tomGFR (Range)	SD ofMeanbias	Accuracy within	
						10% (%)	30% (%)
	^99m^Tc DTPA	77.1±16.3	–	–	–	–	–
All (n = 108)	eGFR_CG_	83.4±20.0	6.3$,¶	5.7 (−40.4–62.9)	20.8	25.0	72.2
	eGFR_MDRD_	71.9±14.5	−5.2¶	−5.1 (−43.9–30.0)	15.8¶	39.8	83.3
	eGFR_CKD-EPI_	76.9±21.2	−0.1#,$	1.9 (−60.3–45.0)	22.9$	26.9	67.6
	^99m^Tc DTPA	101.7±7.9	–	–	–	–	–
≥90 (n = 23)	eGFR_CG_	97.3±20.0	−4.4	−5.9 (−40.4–31.2)	21.9	30.4	78.3
	eGFR_MDRD_	81.4±15.6*	−20.4	−16.2 (−43.9–5.6)	17.2	34.8	65.2
	eGFR_CKD-EPI_	84.9±24.2*	−16.8	−17.9 (−60.3–26.6)	26.5	26.1	65.2
	^99m^Tc DTPA	70.4±10.5	–	–	–	–	–
<90 (n = 85)	eGFR_CG_	79.7±18.4*	9.2$	9.1 (−35.6–62.9)	19.6	23.5	70.6$
	eGFR_MDRD_	69.4±13.1	−1.03*	−2.9 (−30.6–30.0)	12.6¶	41.2	88.2*,¶
	eGFR_CKD-EPI_	74.8±19.9	4.4	5.6 (−40.8–45.0)	19.7$	27.1	68.2$

mGFR, measured glomerular filtration rate,^ 99m^Tc DTPA, technetium-diethylenetriamine pentaacetic acid, 24 hr urine-CrCl, creatinine clearance; eGFR_CG_, Cock-Croft Gault; eGFR_MDRD_, Modification of Diet in Renal Disease; eGFR_CKD-EPI_, chronic kidney disease-Epidemiology collaboration.

* P<0.05, vs. mGFR,

# P<0.05 vs. eGFR_CG_,

$ P<0.05, vs. eGFR_MDRD_,

¶ P<0.05 vs eGFR_CKD-EPI._

### Comparison of Each Equation’s Performance According to mGFR Level after Kidney Donation

We analyzed the performance of equations according to renal function in post-donation cohort (Table 2). In 23 subjects who showed normal renal function (mGFR ≥90 mL·min^−1^·1.73 m^−2^), both eGFR_MDRD_ (P<0.001 vs. mGFR) and eGFR_CKD-EPI_ (P<0.05 vs. mGFR) significantly underestimated mGFR and bias was minimal in eGFR_CG_ (P = 0.74 vs. mGFR). In precision, however, eGFR_MDRD_ (SD of mean bias: 17.2) showed superior value as compared to eGFR_CKD-EPI_ (SD of mean bias: 26.5) (P<0.05) and superior tendency compared to eGFR_CG_ (SD of mean bias : 21.9) (P = 0.110). In 85 subjects who showed decreased renal function (mGFR <90 mL·min^−1^·1.73 m^−2^), eGFR_MDRD_ demonstrated the least bias (P = 0.95 vs. mGFR) and the highest precision (SD of mean bias: 12.6) among three equations but only eGFR_CG_ significantly overestimated mGFR (P<0.01 vs. mGFR). No significant differences in accuracy were detected in any pairs of comparisons in all equations in both groups with normal and decreased renal function (P>0.05, respectively).

### Comparison between Pre-donation and Post-donation Performance of Each Equation

For each individual equation, we compared the performance value between pre-donation and post-donation in 108 patients who took ^99m^Tc-DTPA clearance (mGFR) before and after kidney donation (Table 3). In this analysis, eGFR_MDRD_ showed overall improved performance at post-donation. Precision significantly improved after donation (P<0.001) and the values of bias and accuracy were similar between pre- and post-donation. In contrast, eGFR_CG_ and eGFR_CKD-EPI_ showed overall inferior performance at post-donation compared to pre-donation. Bias from mGFR significantly increased in eGFR_CG_ (P<0.001) and precision significantly decreased in eGFR_CKD-EPI_ (P<0.05) and both equations showed inferior accuracy at post-donation as compared to pre-donation.

**Table 3:** Comparison of the bias, precision and accuracy of each equation to estimate mGFR between before and after kidney donation.

		Mean difference to	Median	SD of mean bias	Accuracy within	
		mGFR			10%(%)	30%(%)
eGFR_CG_	Before	−0.73*	−2.9 (−52.0–76.7)	22.9	33.3*	86.0*
	After	6.3	5.7 (−40.4–62.9)	20.8	25.0	72.2
eGFR_MDRD_	Before	−9.6	−9.0 (−69.1–50.8)	20.8*	35.3	84.1
	After	−5.2	−5.1 (−43.9–30.0)	15.8	39.8	83.3
eGFR_CKD-EPI_	Before	−1.6	0.4 (−55.5–45.1)	19.1	40.6*	91.8*
	After	−0.1	1.9 (−60.3–45.0)	22.9	26.9	67.6

* P<0.05, vs. after donation, eGFR_CG_, Cock-Croft Gault; eGFR_MDRD_, Modification of Diet in Renal Disease; eGFR_CKD-EPI_, chronic kidney disease-Epidemiology collaboration.

### Bland and Altman Plots

**Figure 1:**
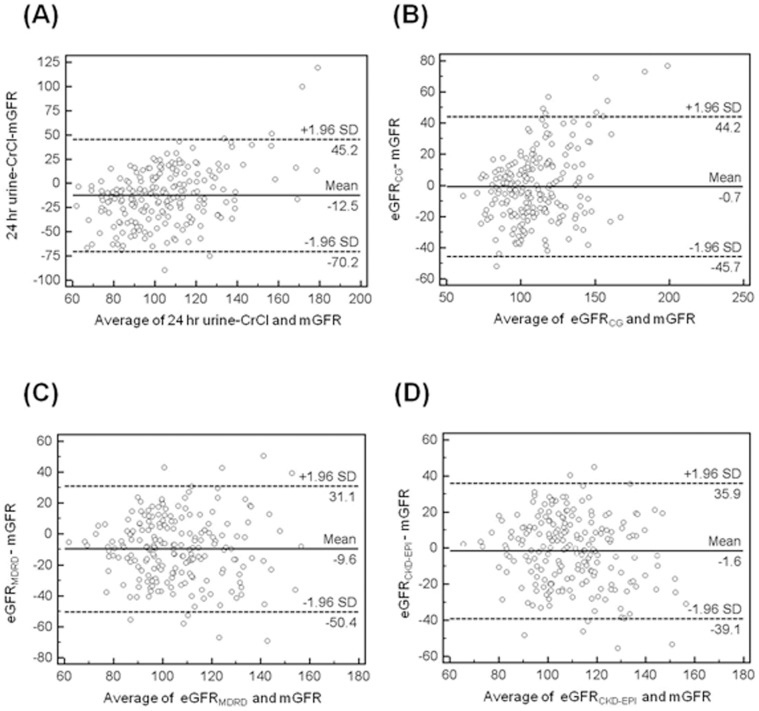
Bland-Altman plots at pre-donation showing the distribution of errors in estimation of measure GFR with eGFR when a given eGFR value is observed. (A) 24 hr urine-CrCl, (B) eGFR_CG_ (C) eGFR_MDRD_ (D) eGFR_CKD-EPI_ mGFR, measured glomerular filtration rate, 24 hr urine-CrCl, creatinine clearance; eGFR_CG_, Cock-Croft Gault; eGFR_MDRD_, Modification of Diet in Renal Disease; eGFR_CKD-EPI_, chronic kidney disease-Epidemiology collaboration.

**Figure 2:**
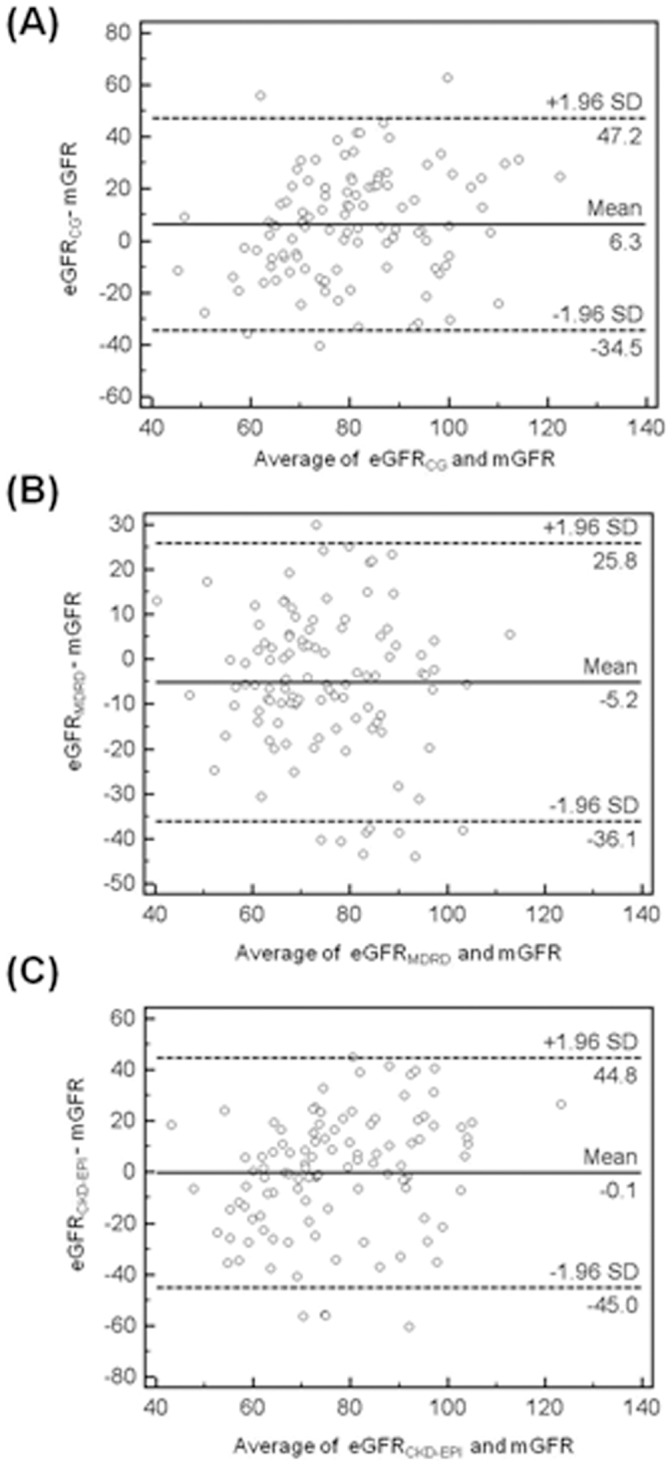
Bland-Altman plots at post-donation showing the distribution of errors in estimation of measure GFR with eGFR when a given eGFR value is observed. (A) eGFR_CG_ (B) eGFR_MDRD_ (C) eGFR_CKD-EPI_ mGFR, measured glomerular filtration rate, eGFR_CG_, Cock-Croft Gault; eGFR_MDRD_, Modification of Diet in Renal Disease; eGFR_CKD-EPI_, chronic kidney disease-Epidemiology collaboration.

The differences between each eGFR and mGFR were illustrated using a graphic technique developed by Bland and Altman. These figures display the span between +1.96 and −1.96 SD of the mean difference (limit of agreement), which represents 95% CI. Before kidney donation, a smaller limit of agreement was found for the eGFR_CKD-EPI_ (37.5) in comparison with the eGFR_MDRD_ (40.7), eGFR_CG_ (44.9), and 24 hr-CrCl (57.7) (Figure 1A–D). However, eGFR_MDRD_ (31.0) showed a smaller limit of agreement than eGFR_CG_ (40.9) and eGFR_CKD-EPI_ (44.9) after kidney donation (Figure 2A–C).

## Discussion

This study investigated the performance of formulas for estimating mGFR in both the pre-donation state (healthy adult) and the post-donation state (uninephric donors). In this study, as compared to other equations, eGFR_CKD-EPI_ showed superior performance in healthy donors, the pre-donation state. In contrast, its performance at predicting mGFR was worse than that of eGFR_MDRD_ in uninephric donors, and the inferiority was more significant in subjects with reduced renal function.

At the pre-donation state, 24 hr-urine CrCl and eGFR_MDRD_ significantly underestimated mGFR, but eGFR_CKD-EPI_ showed only minimal bias. The SD of mean bias was lowest in eGFR_CKD-EPI,_ which suggests the highest precision of this equation. The percentage within 30% of mGFR was significantly higher in eGFR_CKD-EPI_ than in other equations_,_ which suggests the superior accuracy of this equation compared to other equations. This result is fully consistent with the previous reports. [6], [7], [16], [17].

The better performance of eGFR_CKD-EPI_ was more significant when we only included subjects with normal renal function. As reported previously, trends of mGFR underestimation were found in 24 hr-urine CrCl, eGFR_MDRD_, and eGFR_CG_, but only eGFR_CKD-EPI_ showed minimal bias in subjects with normal GFR in this study. [17], [18] In precision and accuracy, eGFR_CKD-EPI_ was superior to the other 3 equations as well for that patient group. However, in subjects with reduced renal function, this better performance was not dominant. This discrepancy of performance according to renal function level may result from differences in the process of equation development. eGFR_MDRD_ and eGFR_CG_ were developed based on CKD patients with reduced renal function, but eGFR_CKD-EPI_ was not specifically developed for that patient population. [4], [7] Indeed, it was previously reported that performance was similar between eGFR_CKD-EPI_ and eGFR_MDRD._ in CKD patients. [19].

At post-donation state, we directly compared the performance between eGFR_CKD-EPI_ and eGFR_MDRD_, and our results showed that eGFR_CKD-EPI_ was inferior to eGFR_MDRD_ in overall performance. eGFR_CKD-EPI_ showed less bias compared to eGFR_MDRD._/But as shown in high SD of mean difference between mGFR and eGFR_CKD-EPI_, which suggests low precision, the difference from mGFR was distributed widely in both the positive and negative directions. Negatively and positively biased values may offset each other during the calculation of mean value and may have resulted in the minimal bias of eGFR_CKD-EPI_. In another performance such as,precision and accuracy, eGFR_CKD-EPI_ showed inferior performance compared to eGFR_MDRD_. In addition, eGFR_MDRD_ showed better or similar performance at post-donation compared to pre-donation, but eGFR_CKD-EPI_ showed inferior performance at post-donation compared to pre-donation within individual analysis.

The reason for the superior performance of eGFR_MDRD_ at post-donation state is unclear. One possible reason is that the proportion of subjects with reduced renal function was greater in this group compared to the pre-donation group. Indeed, the performance of eGFR_MDRD_ is not inferior to eGFR_CKD-EPI_ in subjects with reduced renal function in pre-donation cohort. But it cannot explain the better performance of the eGFR_MDRD_ than eGFR_CKD-EPI_ in post-donation cohort with normal renal function. Hence, the more important reason may bethe specific situation of uninephric kidney donors, which is different not only from healthy populations but also from patients with chronic kidney disease. In these subjects, removal of 1 kidney leads to a subsequent reduction in GFR without disease-associated changes in body composition. [17] Renal tissue reduction is accompanied by compensatory hyperfiltration by the remaining nephrons with increases in single-nephron GFR. [20], [21] Therefore, the renal function only showed a modest decrease compared to its level before KT because of the remaining kidney’s hyperfiltration. In this specific condition, the performance of estimating equations in those patients may show different pattern compared to healthy populations or chronic kidney disease state.

Another possible reason is that the performance of equations for estimating GFR could be affected by the demographic and ethnic factors. Most estimating equations developed primarily based on western populations, hence they may show different performance when used in Asian because of the significant anthropometric difference. Of note, many studies about the performance of estimating equations conducted on Asian showed different outcomes compared to the result from Western populations. [22], [23], [24] For those reasons, it has been reported that modification is necessary in the use of eGFR_CKD-EPI_ on multiethnic Asian populations. [19], [25] But clear conclusion about this issue in Korean may need further investigation.

It is interesting that eGFR_CG_ seems to be nearly unbiased in the potential kidney donors. Because the eGFR_CG_ was derived to estimate creatinine clearance, which is known to overestimate mGFR by 10% to 20% as a result of creatinine secretion, this may be interpreted as a fortuitous cancellation of errors. [14], [26] eGFR_CG_ apparently underestimated creatinine clearance by 10% to 20%, thus producing a mean value close to the mean mGFR. In addition, some previous reports indicated that eGFR_CG_ is more appropriate than eGFR_MDRD_ in subjects without kidney disease. [23], [27] Therefore, it is possible that eGFR_CG_ showed superior accuracy and less bias compared to eGFR_MDRD_ before kidney donation. After donation, however, when a significant portion of subjects showed reduced renal function, the overall performance of eGFR_CG_ was inferior to eGFR_MDRD,_ as expected.

Usually, CrCl using 24-hour urine collection is not recommended for the estimation of renal function because of the possibility of urine loss during collection, which can cause an inaccurate result. In addition, this method is so inconvenient for patients compared to other methods. [12] Indeed, 24 hour urine was adequately collected only in 31.9% of total donors according to normal range of creatinine excretion. [28] Hence the inaccuracy of 24-hour urine CrCl in the estimation of renal function, including the underestimation of mGFR, may not result from its own low performance but from the inadequate urine collection. Therefore, 24 hr-urine CrCl may not be appropriate for the estimation of GFR before or after donation considering the difficulty of adequate urine collection and patient’s convenience.

This study does have some limitations. We could not use the inulin clearance, the gold standard method for measuring true GFR. However, ^99m^Tc-DTPA clearance is relatively less biased and has been accepted as the accurate method for the measurement of GFR in previous reports. [10], [29], [30], [31] Second, this is a retrospective single-center study, which only included Korean adults. Therefore, the results of this study may not be definite in Western populations which have different anthropomorphic characteristics. To apply our results in those populations, further investigation may be required. Third, eGFR_MDRD_ showed tendency to overestimate the prevalence of CKD, as shown in a previous report, which means that this estimating equation must be used with some caution in the follow-up of uninephric donors. [32].

Nevertheless, this study differs from previous studies in that we used unified and standard methods to measure Scr. A weak point detected in many previous reports was that the Scr assay was either not standardized or not unified, hence the need for a calibration process, which could induce some bias in the results. [17], [32] It is possible that the divergences in Scr determination and calibration may have accounted for the heterogeneity of the results in previous studies. [14], [33], [34] To overcome it, we only included subjects who were tested with isotope dilution mass spectrometry (IDMS)-traceable creatinine, which helps to estimate GFR more accurately. [35].

In conclusion, in the potential kidney donor, eGFR_CKD-EPI_ showed better performance than other GFR estimating equations including eGFR_MDRD_ in the prediction of renal function. However, in the uninephric state after kidney donation, the overall performance of eGFR_CKD-EPI_ was inferior to eGFR_MDRD_, which suggests that the eGFR_MDRD_ is more appropriate for the estimation of renal function during follow-up of uninephric kidney donors.
